# Late effects of high-dose methotrexate in childhood cancer survivors: a Swiss single centre observational study

**DOI:** 10.1007/s12672-024-00861-0

**Published:** 2024-01-25

**Authors:** Kevin Brunold, Maria Otth, Katrin Scheinemann

**Affiliations:** 1 Faculty of Medicine, University of Zurich, Zurich, Switzerland; 2 Faculty of Health Sciences and Medicine, University of Lucerne, Lucerne, Switzerland; 3 Division of Oncology-Haematology, Children’s Hospital of Eastern Switzerland, St Gallen, Switzerland; 4 Department of Oncology, University Children’s Hospital Zurich, Zurich, Switzerland; 5 Department of Pediatrics, McMaster Children’s Hospital and McMaster University, Hamilton, ON Canada

## Abstract

**Importance:**

Childhood cancer survivors (CCS) are at risk for late effects of different organ systems. The currently available screening recommendations for those treated with high-dose methotrexate (HD-MTX) are not uniform and the available literature is limited.

**Objective:**

We aim to assess the prevalence and severity of late effects in CCS treated with HD-MTX at a single centre in Switzerland. We focus on organ systems defined at risk by the long-term follow-up care guidelines of the children’s oncology group (COG), because this guideline has a holistic approach, is evidence based, and up to date.

**Methods:**

We used the modified Common Terminology Criteria for Adverse Events (CTCAE) to assess late effects in 15 different organ systems. Eligible were CCS diagnosed with cancer younger than 18 years and treated with HD-MTX, defined as at least 1 g per body surface area (≥ 1 g/m^2^).

**Results:**

We analysed 32 CCS with a median follow-up of 12.1 years. The endocrine system was most frequently affected by adverse events (69%), followed by the musculoskeletal (57%) and neuropsychological (38%) systems. The hepatobiliary (9%) and immunological (6%) systems were the least affected ones. Within the endocrine system, overweight/obesity was the most frequent and severe diagnosis.

**Conclusion:**

Late effects in CCS treated with HD-MTX are frequent. Our findings could add to the COG guidelines, where only screening for the musculoskeletal, neuropsychological, and hepatobiliary systems are recommended. More patient data need to be collected and analysed using the suggested standardised approach, to increase the quality of evidence for future screening recommendations.

**Supplementary Information:**

The online version contains supplementary material available at 10.1007/s12672-024-00861-0.

## Introduction

Around 350 children, adolescents, and young adults below the age of 20 years are newly diagnosed with cancer in Switzerland every year [1]. Improvements within the field of paediatric oncology have greatly increased the survival rates of these patients over the past decades [2, 3], resulting in a growing number of childhood cancer survivors (CCS) [4, 5]. Studies show that over 70% of these patients develop chronic medical conditions, referred to as late effects or adverse events, caused by the cancer or its treatment later in their lives [6–8]. To detect these late effects early, several different national and international long-term follow-up (LTFU) care guidelines have been established. These guidelines also include screening recommendations following the treatment with methotrexate, but they are not uniform (Additional file 1: Table S1) [9–11]. Screening for reduced bone mineral density (BMD) in CCS after methotrexate (MTX) is for example recommended in the LTFU care guidelines from the Children’s Oncology Groupe (COG), is listed in the guideline from the UK with a question mark, not mentioned in the Dutch guidelines, and not included in the recommendation from the international Guideline Harmonization Group (IGHG) due to no significant associations between low BMD and treatment with MTX [9–12]. This difference in recommendations might be because the current literature and quality of evidence on late effects after childhood cancer treatment with high-dose methotrexate (HD-MTX) is very limited [13].

Methotrexate is a folate antagonist, commonly used in the treatment of different childhood cancers, such as acute lymphoblastic leukaemia (ALL), non-Hodgkin lymphoma, osteosarcoma, or in certain central nervous system tumours [14–16]. The most commonly used definition of HD-MTX is an intravenous dose of at least 1 g per body surface area (≥ 1 g/m^2^), because from this dose onwards leucovorin rescue is indicated [17]. The aim of this study is to assess the prevalence and severity of late effects in CCS treated with HD-MTX at a single institution in Switzerland, the Kantonsspital Aarau (KSA). By applying a standardised approach and using the modified Common Terminology Criteria for Adverse Events (CTCAE) [18, 19], we aim to provide data that can be used for future LTFU care recommendations.

## Methods

For this single centre observational study, data were collected within the framework of the Young Survivors registry [20], which was approved by the cantonal ethics committee EKNZ (Ethikkommission Nordwest- und Zentralschweiz; AO_2020–00012) and was conducted in accordance with the guidelines of the EKNZ and the 1964 Declaration of Helsinki and its later amendments. Written informed consent to participate was obtained from all individual participants or their legal representatives or parents. The Young Survivors registry has a longitudinal setup with a retrospective and a prospective design. The study population includes children and adolescents who have been diagnosed with cancer younger than 18 years, completed their treatment, and have entered follow-up care. For this study, all cancers were diagnosed between 2000 and 2010, allowing for a follow-up time of at least 10 years. To be included, patients must have been treated with HD-MTX and have given consent to participate in the Young Survivors registry or signed the general consent at KSA. Additionally, we included retrospective data of CCS under the absence of informed consent, if they were either deceased or if it would have been a disproportionate effort to find a current address, according to the Human Research Act, HFG Art. 34.

Childhood cancer survivors treated at the KSA receive a survivorship care plan, containing detailed information about the diagnosis, the treatment received, potential late effects, and respective screening recommendations for the organ systems at risk, based on the COG LTFU care guidelines [9]. We decided to use the COG LTFU care guidelines to make the cohort comparable to others. In addition, this guideline covers all organ systems, whereas the IGHG guidelines exist for separate organ systems and do not cover all. Age at diagnosis and data on diagnosis, treatment, and organ systems at risk for late effects were collected from the survivorship care plans. Information on organ function was collected manually in annual intervals since treatment completion from the electronic medical records [20]. Data on organ function and different organ systems was gathered by using the modified CTCAE criteria [18] and contained information about the occurrence and severity of late effects in 15 different organs systems (auditory, cardiovascular, endocrine, gastrointestinal, hepatobiliary, haematologic, immunologic, infectious, pulmonary, musculoskeletal, neurologic, neurocognitive, renal/urinary, reproductive/genital and ocular/visual). For HD-MTX, the COG LTFU care guidelines recommend screening for reduced BMD, hepatic dysfunction, and neurocognitive deficits [9]. All data were entered manually into the electronic database (REDcap [21, 22]) of the Young Survivors registry. This process was done by one author (KB) and verified by another (MO).

Stata (StataCorp. 2021. Release 17. College Station, TX: StataCorp LLC) was used to extract and analyse the patient data from the electronic database and apply descriptive statistics. The primary outcomes were prevalence and severity of late effects according to the modified CTCAE criteria in different organ systems. Because most patients were diagnosed with ALL and received approximately the same dose of HD-MTX, with only few CCS who received lower (e.g., non-Hodgkin Lymphoma) or higher (e.g., osteosarcoma) doses, no regression analysis could be carried out to determine the impact of the dose of HD-MTX. Additionally, due to the large difference in the number of CCS per cancer type, we did not perform stratified analysis by cancer type nor multivariate analysis.

Due to clinical relevance, we specified the definition of two outcomes—arterial hypertension and abnormal blood count. For adults, arterial hypertension grade I according to the modified CTCAE criteria (systolic 120-139 mmHg / diastolic 80–89) corresponds to the clinical definition of prehypertension and the modified CTCAE grade II arterial hypertension corresponds to the clinical definition of stage one hypertension (systolic 140–159 mmHg / diastolic 90-99 mmHg). We therefore included in adults only arterial hypertension grade II and higher according to the modified CTCAE criteria. To distinguish low blood counts due to treatment-induced acute toxicity from other reasons, we differentiated this outcome into low blood counts within and more than 5 years after treatment completion.

## Results

**Fig. 1:**
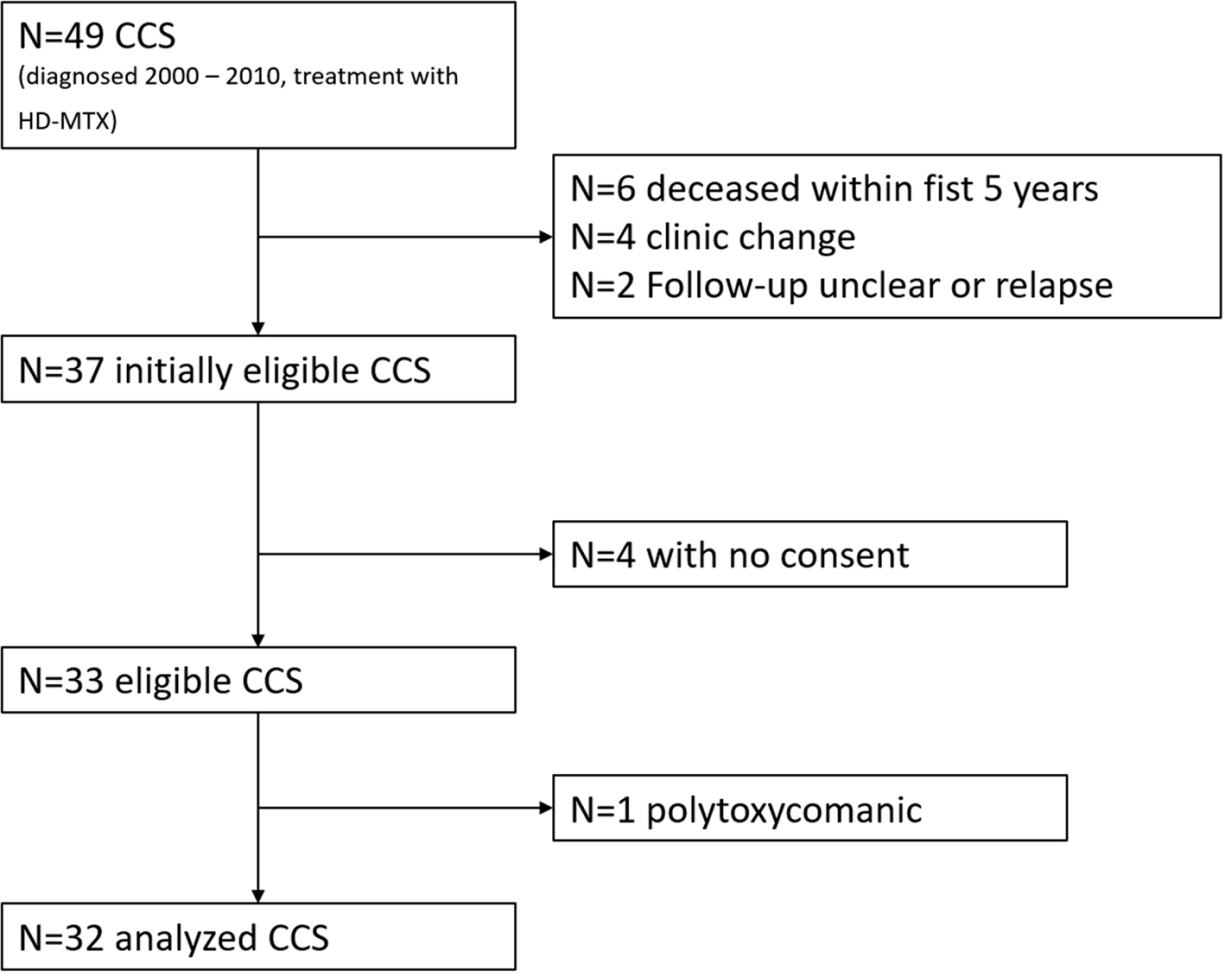
Patient tree

Between 2000 and 2010, 49 children and adolescents below 18 years of age were treated with HD-MTX. Of those, 32 CCS were eligible (65%). The main reasons for exclusion were death within the first five years (n = 5), change of treating clinic (n = 4), and missing informed consent (n = 4) (Fig. 1).

Slightly more CCS were male (56%) with leukaemia (78%) being the most frequent primary cancer diagnosis, followed by malignant bone tumours (13%) and lymphoma (9%) (Table 1). All 32 CCS received multiagent chemotherapy containing HD-MTX, 14 (44%) additional radiotherapy, six (19%) had to undergo surgery, and four (13%) received haematopoietic stem cell transplantation (HSCT). Due to the protocols used, intrathecal MTX was standard of care for all CCS diagnosed with ALL. The median age at primary cancer diagnosis was 6.37 years (IQR 3.48–12.69) with a median treatment duration of 2.1 years (IQR 1.99–2.12.). The median follow-up time after treatment completion was 12.1 years (IQR 11.05–16.61) (Table 1).

**Table 1:** Patient characteristics (n = 32)

	n (%)
Sex, male	18 (56)
Primary diagnosis	
Leukemia	25 (78)
Malignant bone tumours	4 (13)
Lymphoma	3 (9)
Age at primary cancer diagnosis, (median, IQR) [years]	6.37 (3.48–12.69)
Relapse	3 (9)
Age at relapse, (median, IQR) [years]	7.93 (1.98–19.26)
Secondary malignancy	0
Chemotherapy	32 (100)
Radiotherapy	14 (44)
Surgery	6 (19)
HSCT	4 (13)
Central venous line	32 (100)
Age at first visit, (median, IQR) [years]	8.5 (5.73–14.75)
Age at last visit, (median, IQR) [years]	23.42 (19.95–26.17)
Time from diagnosis to first visit (median, IQR) [years]	2.04 (1.99–2.12)
Time from diagnosis to last visit at LTFU care clinic (median, IQR) [years]	15.59 (12.40–18.64)
Time from first to last visit at LTFU care clinic (median, IQR) [years]	12.1 (11.05–16.61)

All CCS presented with at least one adverse event according to the modified CTCAE criteria once during their follow-up time. Half of the CCS (n = 16) presented with five or more adverse events at least once, with a maximum of 10 adverse events in one CCS. Slightly more CCS (n = 19, 59%) presented at least once with a severe adverse event, defined as grade 3–5 according to the modified CTCAE criteria.

All CCS were at risk for neurocognitive deficits, impaired liver function, and reduced BMD. Of those, the musculoskeletal and neuropsychological system ranked among the highest affected organ systems (rank two and three) in our population (Table 2**, **Additional file 1: Table S2). Half of CCS (56%) presented with musculoskeletal deficits and one third (38%) with neurocognitive deficits at least once during their follow-up time. Within the musculoskeletal system, reduced BMD was the most frequent diagnosis with 11 out of the 18 CCS affected by musculoskeletal outcomes. Ten of these CCS were treated with steroids, also associated with a reduced BMD [23]. Of the neuropsychological deficits, attention was the most frequently affected domain with 6 affected CCS, followed by depression (n = 4), and executive function deficit (n = 4) (Table 2, Additional file 1: Table S2).

**Table 2:** Organ systems at risk according to COG guidelines, frequency of events, and affected organ system with the most frequent outcome

No of patients at risk (COG guidelines)	No of patients with events	Organ system, most frequent outcome
15 (46.8%)	22 (68.75%)	Endocrine Overweight / obesity (n = 17)
32 (100%)	18 (56.25%)	Musculosceletal Bone mineral density deficit (n = 11)
32 (100%)	12 (37.5%)	Neuropsychological Attention deficit (n = 6)
32 (100%)	9 (28.13%)	Cardiovascular (without prehypertension) Clinical hypertension (n = 8)
32 (100%)	9 (28.13%)	Neurologic Headache (n = 6)
3 (9.38%)	9 (28.13%)	Infectious Gastrointestinal & genitourinary infection (n = 3 each)
4 (12.5%)	6 (18.75%)	Auditory Hearing loss (n = 5)
29 (90.63%)	6 (18.75%)	Ocular Reduced visual acuity (n = 6)
4 (12.5%)	5 (15.63%)	Gastrointestinal Gastritis / duodenitis (n = 4)
4 (12.5%)	5 (15.63%)	Pulmonary Asthma (n = 3)
32 (100%)	5 (15.63%)	Reproductive Dyspareunia/ Leydig cell insufficiency/ Primary ovarian failure/ AMH outside normal range/ Inhibin B outside normal range (n = 1 each)
32 (100%)	5 (15.63%)	Renal Chronic kidney disease (n = 4)
32 (100%)	5 (15.63%)	Hematologic (> 5 years after finishing treatment) Anaemia (n = 3)
32 (100%)	3 (9.38%)	Hepatobiliary Hepatopathy/alanine and aspartate aminotransferase increased (n = 3)
3 (9.38%)	2 (6.25%)	Immunologic Graft-versus-host disease, immunodeficiency (n = 1 each)

Three CCS presented with an adverse event of the hepatobiliary system. With two CCS, the immunological system was affected the least frequent. The endocrine system was the most frequently affected organ system with 22 CCS (69%) presenting at least once with an endocrinological adverse event during their follow-up time. Overweight/obesity was the most frequent diagnosis with 17 out of the 22 affected CCS (Table 2). Eight of these 17 CCS presented with Grade 3 obesity, resulting in the most frequent severe medical condition recorded in this cohort. A common denominator within these 17 CCS was the treatment with steroids, which was administered to 16 of them.

Due to the use of anthracyclines, all CCS in this cohort were defined at risk for cardiovascular outcomes. After exclusion of the 18 CCS with prehypertension, nine CCS (28%) presented at least once with a cardiovascular outcome (Table 2). This ranks the cardiovascular system at number four of the affected organ systems, together with neurological and infectious medical conditions. With eight out of these nine CCS, the most frequent diagnosis was hypertension (systolic 140 mmHg / diastolic ≥ 90 mmHg), defined as Grade 2 or higher.

After the exclusion of 16 CCS with haematologic adverse events within the first five years after treatment completion, five CCS (16%) presented with long lasting haematological medical conditions, including three with anaemia and two with thrombocytopenia (Additional file 1: Table S3). Five CCS presented at least once with a medical condition of the pulmonary, gastrointestinal, renal, and reproductive systems. The ocular and auditory systems were affected in six cases each (19%) (Table 2 Additional file 1: Table S2).

## Discussion

All CCS in our cohort presented at least once with an adverse event according to the modified CTCAE criteria with a median follow-up time of 12 years. Two out of three organ systems (musculoskeletal, neuropsychological) defined at risk by the COG LTFU care guidelines did rank within the top three of the most frequently affected organ systems in our cohort. The third organ system defined at risk based on the COG LTFU care guidelines, the hepatobiliary system, ranked second lowest (n = 3, 9%) in our cohort. With 22 CCS (69%), the endocrine system was most frequently affected, with overweight/obesity being the most frequent outcome. It was also the organ system with the highest number of CCS with a severe adverse event.

The systematic review by Daetwyler et al. shows that CCS treated with HD-MTX seem not to be at a higher risk for significantly lower BMD than controls [13]. Lequin et al. assessed bone health using a spinal dual-energy X-ray absorptiometry (DXA) in long-term survivors of ALL [24]. Nine out of 21 children (43%) had a standard deviation score below zero for the lumbar spine and ten (48%) for total body. Tillman et al. evaluated BMD as a ratio between bone mineral content and bone area, both assessed using DXA [25]. The areal BMD of total body and lumbar region were not different between leukaemia survivors and controls, but the mean lumbar volumetric BMD of survivors was significantly lower. Also van der Sluis et al. used DXA to assess BMD in 23 leukaemia survivors [26]. The mean standard deviations for BMD of the lumbar spine and total body were within the normal range and no survivors had a BMD below -2. All these results are comparable with the findings in our cohort with 34% CCS (n = 11) diagnosed with a reduced BMD. These results highlight the different approaches how BMD results are measured and analysed. Therefore, comparison of results is only possible to a limited extent. A standardized approach on how to assess bone health, to collect the data, and to analyse them is needed in the future and is partly covered in the IGHG recommendation [12].

Several studies have shown that next to HD-MTX, the treatment with steroids is a major risk factor for a reduced BMD [27–29]. The IGHG recommendation goes in the same direction, where surveillance is recommended in CCS receiving corticosteroids as part of their anti-cancer treatment, but not for those with MTX [12]. In our cohort, ten out of eleven CCS with a reduced BMD have received both drugs. Based on our data, we cannot distinguish the impact of HD-MTX and steroids on BMD, whether there is an additive or potentiated effect, or whether the reduced BMD was solely a result of the steroids.

Similar to reduced BMD, some studies report an association between the treatment with steroids and the diagnosis of overweight/obesity in CCS [30, 31]. Half of the CCS in our cohort (n = 17; 53%) were overweight/obese, including eight CCS with Grade 3 obesity. This is higher than in the Swiss general population [32] with 22.4% of male and 11.6% of female adolescents and young adults aged 15–24 years being overweight and 5.7% and 5.2% being obese respectively. In Swiss children aged 6–12 years, 11.7% were overweight and 3.3% obese based on results from the Swiss monitoring system for addiction and non-communicable diseases in 2017, with no difference between boys and girls [33]. Again, the available data did not allow to analyse for potential causality in our cohort. One could speculate that due to the high proportion of affected CCS treated with high-dose steroids and the currently missing evidence of a correlation with HD-MTX and overweigh/obesity, the impact of steroids seems much more likely to be the cause of this recorded adverse event.

The impact of methotrexate on neurocognitive, neurobehavioral, and brain imaging outcomes is widely discussed, especially since the avoidance of cranial radiotherapy in most patients with ALL. Despite the difficulty to assess the impact of a single agent in patients receiving multi-agent chemotherapy, pharmacodynamic studies show an association between higher MTX plasma concentration and executive dysfunction as well as imaging changes in regions responsible for executive function [34]. Acute MTX-induced leukoencephalopathy is a risk factor for long-term neurobehavioral problems [35]. The theory has emerged that higher doses and an earlier start of folinic acid (FA) might reduce MTX-induced neurotoxicity [36]. A non-systematic review including neuropsychological studies and assessing the correlation between neuropsychological outcomes and FA rescue indicates that cohorts with lower FA doses have more frequent neuropsychological outcomes than cohorts with higher FA doses [37]. However, increasing the FA dose or shortening the interval between MTX and FA might reduce the cure rate [38]. This aspect needs further investigations, also taking the genetic aspect into account, as Mateos et al. identified single-nucleotide polymorphism associated with MTX neurotoxicity [39]. The impact of genetic factors and polymorphism becomes increasingly relevant for the development and clinical course of late effects. Certain data are available for ototoxicity and platinum agents [40]. Genetic testing to assess the susceptibility for late effects is currently not standard of care and not part of the Young Survivors registry. However, if such data are available at later stages, the registry would be a great clinical source.

The strength of this study is the standardised and systematic approach to assess and categorize medical conditions in CCS. The data were entered into the electronic registry database by using the grading system of the modified CTCAE criteria. Through the control of the entered data by a second author, the risk of wrong data entry was kept to a minimum. The retrospective design of data collection is a limitation, linked to the risk of missing data due to incomplete medical records and that tests might not have been performed in earlier years. Further, the heterogeneous cohort with respect to the diagnosis and the impact of other chemotherapeutic agents or complications that may have caused the reported outcomes cannot be excluded. Additionally, the awareness about possible late effects and therefore the focus of the annual screening visits at KSA has changed over time, leading to potential time bias. A further limitation is the small cohort, linked to its single centre design. Consequently, further statistical analyses, e.g. multivariate analysis, to control for certain confounders or to compare different patient groups could not be carried out.

## Conclusion

CCS treated with HD-MTX are at risk for late effects in various organ systems. While the musculoskeletal and neuropsychological organ systems were among the highest affected, the hepatobiliary system was the organ system with the second lowest number of affected CCS. These findings differ from the COG guidelines, where all three organ systems are defined at risk in CCS treated with HD-MTX and screening is recommended. This highlights the need for more data to update LTFU care recommendations following the exposure to HD-MTX. To increase the power, multicentre studies are needed where the data should be collected in a standardized way to allow data collection in different centres, in different countries, and longitudinally.

### Supplementary Information

Additional file1 **Annex 1.** Comparison of organ systems at risk due to high-dose methotrexate and screening recommendations listed by long-term follow-up care guideline. **Annex 2.** Table of detailed CTCAE adverse events overall, at least once per survivor during follow-up, and during the last visit. **Annex 3.** Haematological adverse events less and more than 5 years following completion of treatment. (DOCX 47 KB)

## Data Availability

The data are available from the authors upon reasonable request.
