# Supplementary material for: Late effects of high-dose methotrexate in childhood cancer survivors: a Swiss single centre observational study
Source: Discov Oncol. 2024 Jan 25;15:17. doi: 10.1007/s12672-024-00861-0 (PMC10810765; doi:10.1007/s12672-024-00861-0)
Supplement: Supplementary file 1 — Additional file1 Annex 1. Comparison of organ systems at risk due to high-dose methotrexate and screening recommendations listed by long-term follow-up care guideline. Annex 2. Table of detailed CTCAE adverse events overall, at least once per survivor during follow-up, and during the last visit. Annex 3. Haematological adverse events less and more than 5 years following completion of treatment. (DOCX 47 KB) [file 12672_2024_861_MOESM1_ESM.docx]

**Late effects of high-dose methotrexate in childhood cancer survivors – a Swiss single centre observational study**

Discover Oncology - Springer

Kevin Brunold^1,2^, Maria Otth^2,3,4^, Katrin Scheinemann^2,3,5^

^1^ Faculty of Medicine, University of Zurich, Zurich, Switzerland

^2^ Faculty of Health Sciences and Medicine, University of Lucerne, Lucerne, Switzerland

^3^ Division of Oncology-Haematology, Children's Hospital of Eastern Switzerland, St

Gallen, Switzerland

^4^ Department of Oncology, University Children’s Hospital Zurich, Zurich, Switzerland

^5^ Division of Pediatric Hematology/Oncology, McMaster Children’s Hospital and McMaster

University, Hamilton, ON, Canada

Corresponding author: maria.otth@kispisg.ch

**Supplementary Table 1:** Comparison of organ systems at risk due to high-dose methotrexate and screening recommendations listed by long-term follow-up care guideline

| **Late effect** | **COG, version 5.0** | **UK** | **SKION** | **IGHG** |
| --- | --- | --- | --- | --- |
| Reduced bone mineral density (BMD) | Bone density evaluation (DXA) as baseline at entry into LTFU, repeat as clinically indicated | MTX as indication for screening listed with question mark  Regular history for back pain and fractures; consider DEXA in: BMT recipients, survivors of ALL or medulloblastoma, history of fracture or back pain, history of growth hormone deficiency or hypogonadism | Not mentioned | Not included in recommendation due to no significant associations between low BMD and treatment with MTX |
| Renal toxicity | No known renal late effect | Regularly in LTFU clinic:  1) Measure BP  2) Urinalysis for proteinuria – if positive, measure urine protein:  3) Monitor growth at least annually until final height  4) Serum U+Es / creatinine; GFR only if high creatinine  1-6months post-treatment; repeat 5 yearly if creatinine normal; measure GFR only if high creatinine | Not mentioned | Guideline in development |
| Hepatic dysfunction | Yearly physical examination (scleral icterus, jaundice, ascites, hepato-, splenomegaly)  ALT, AST, Bilirubin at entry into LTFU, repeat as clinically indicated | At end of treatment and regularly in LTFU clinic thereafter, examine for  hepatosplenomegaly and stigmata of chronic liver disease  At end of treatment and as clinically indicated in LTFU clinic thereafter,  measure liver function tests (bilirubin, transaminases, alkaline phosphatase) | At first LATER-out-patient visit: AST, ALT, GGT; repeat if clinically indicated ; testing for HBV and CMV serology if the results are unknown | Surveillance for liver injury is reasonable in CCS with history of MTX; physical examination and liver enzymes (AST, ALT, GGT, ALP) at entry into LTFU, repeat as clinically indicated |
| Neurocognitive deficits | History of education and/or vocational progress yearly  Referral for formal neuropsychological evaluation at entry into LTFU, then periodically as clinically indicated for patient with evidence of impaired educational or vocational progress | Neuropsychological (functional or cognitive impairment): routinely history and examination – memory, attention, intelligence, visual-spatial,  verbal and fine motor function | Specific neuropsychological history (school, work, living situation, social functioning (social contacts, hobbies), mobility (driving license))  “Kaufman Short Neuropsycho-logical Assessment Procedure” (K-SNAP)in CCS following (neurological) complications, e.g., MTX-induced encephalopathy; K-SNAP at first LATER-out-patient visit and every 3 years until 3 normal consecutive tests | Guideline on neurocognitive problems in development |
| Clinical leukoencephalopathy | History of cognitive, motor and/or sensory deficits, seizures, other neurological symptoms yearly  Neurological exam yearly | See “Neurocognitive deficits” | Not mentioned | Guideline on neurocognitive problems in development |
| Neurological deficits |  | Only mentioned in CCS after BMT: routinely history and examination – especially headaches, raised intracranial pressure, cranial nerve and motor function, gait, peripheral nerve function | Not mentioned | Guideline on neurocognitive problems in development |
| Respiratory |  | Only mentioned in CCS after BMT: Routine evaluation needed of:  History and examination – exercise tolerance, smoking ; pulmonary function testing (PFT) ; consider chest x-ray if symptomatic or if PFTs severely abnormal | Not mentioned | Guideline in development |

- Childrens Oncology Group. Long Term Follow-Up Guidelines Version 5.0. 2018. <http://www.survivorshipguidelines.org/pdf/2018/COG_LTFU_Guidelines_v5.pdf>
- United Kingdom Children’s Cancer Study Group. Therapy based long term follow-up - Practice Statement. 2005. <https://www.cclg.org.uk/write/MediaUploads/Member%20area/Treatment%20guidelines/LTFU-full.pdf>
- SKION DCOG. Guidelines for follow-up in survivors of childhood cancer 5 years after diagnosis. 2010. [https://www.skion.nl/workspace/uploads/vertaling-richtlijn-LATER-versie-final-okt-2014_2.pdf. Accessed 28 Aug 2023](https://www.skion.nl/workspace/uploads/vertaling-richtlijn-LATER-versie-final-okt-2014_2.pdf.%20Accessed%2028%20Aug%202023).
- International Guideline Harmonization Group (IGHG): <https://www.ighg.org>

**Supplementary Table 2:** Table of detailed CTCAE adverse events overall, at least once per survivor during follow-up, and during the last visit

| **CTCAE (449 visits): overall** | | | | | **At least once during whole follow-up (n=32)** | | | **Last visit (n=32)** | | |
| --- | --- | --- | --- | --- | --- | --- | --- | --- | --- | --- |
|  | At risk  n (%) | Outcome  n (%) | At risk **&** outcome  n (%) | | At risk  n (%) | Outcome  n (%) | At risk **&** outcome  n (%) | At risk  n (%) | Outcome  n (%) | At risk **&** outcome  n (%) |
| **Neuropsychological and Psychological Health** | 449 (100) | 35 (7.8) | 35 (7.) | | 32 (100) | 12 (37.50) | 12 (37.50) | 32 (100) | 3 (9.38) | 3 |
| Attention deficit |  | Grade 1: n=5  Grade 2: n=2 |  | |  | 6 (18.75)  Grade 1: n=5  Grade 2: n=1 | 6 (18.75) |  | 1 (3.13)  Grade 2: n=1 | 1 |
| Executive function deficit |  | Grade 1: n=4 |  | |  | 4 (12.50)  Grade 1: n=4 | 4 (12.50) |  | 1 (3.13)  Grade 1: n=1 | 1 |
| Fine-motor dexterity deficit |  | Grade 1: n=1 |  | |  | 1 (3.13)  Grade 1: n=1 | 1 (3.13) |  | 0 |  |
| Memory deficit |  | Grade 1: n=6 |  | |  | 3 (9.38)  Grade1: n=3 | 3 (9.3) |  | 0 |  |
| Processing speed deficit |  | Grade 1: n=1 |  | |  | 1 (3.13)  Grade1: n=1 | 1 (3.13) |  | 0 |  |
| Depression |  | Grade 1: n=5  Grade 2: n=2  Grade 3: n=1  Grade 4: n=1 |  | |  | 4 (12.50)  Grade 1: n=1  Grade 2: n=1  Grade 3: n=1  Grade 4: n=1 | 4 (12.50) |  | 1 (3.13)  Grade 2: n=1 | 1 |
| Insomnia |  | Grade 1: n=7 |  | |  | 1 (3.13)  Grade 1: n=1 | 1 (3.13) |  | 0 |  |
| Suicide attempt (past 12 months and lifetime) |  | Grade 4: n=1 |  | |  | 1 (3.13)  Grade 4: n=1 | 1 (3.13) |  | 0 |  |
| Suicide Ideation (past 12 months) |  | Grade 3: n=1 |  | |  | 1 (3.13)  Grade 3: n=1 | 1 (3.13) |  | 0 |  |
| **CTCAE (449 visits): overall** | | | | | **At least once during whole follow-up (n=32)** | | | **Last visit (n=32)** | | |
|  | At risk  n (%) | Outcome  n (%) | | At risk **&** outcome  n (%) | At risk  n (%) | Outcome  n (%) | At risk **&** outcome  n (%) | At risk  n (%) | Outcome  n (%) | At risk **&** outcome  n (%) |
| **Auditory, Hearing** | 48 (10.69) | 42 (9.35) | | 39 | 4 (12.50) | 6 (18.75) | 4 (all at risk) | 4 (12.50) | 3 (9.38) | 3 |
| Cerumen impaction |  | Grade 2: n=14 | |  |  | 1 (3.13)  Grade 2: n=1 | 1 (at risk) |  | 1 (3.13)  Grade 2: n=1 | 1 |
| Hearing loss |  | Grade 1: n=17  Grade 2: n=9  Grade 3: n=3 | |  |  | 5 (15.63)  Grade 1: n=4  Grade 3: n=1 | 4 (all at risk) |  | 2 (6.25)  Grade 1: n=1  Grade 2: n=1 | 2 |
| Tinnitus |  | Grade 1: n=1  Grade 2: n=7 | |  |  | 2 (6.25)  Grade 1: n=1  Grade 2: n=1 | 1 (at risk) |  | 0 |  |
| **Cardiovascular** | 449 (100) | 114 (25.39) | | 114 | 32 (100) | 27 (84.38) | 27 (all at risk) | 32 (100) | 15 (46.88) | 15 (46.8) |
| Dysrhythmia |  | Grade 1: n=1 | |  |  | 1 (3.13)  Grade 1: n=1 | 1 (at risk) |  | 0 |  |
| High total cholesterol |  | Grade 2: n=2 | |  |  | 2 (6.25)  Grade 2: n=2 | 2 (at risk) |  | 1 (3.13)  Grade 2: n=1 | 1 |
| Hypertension in adults |  | Grade 1: 57  Grade 2: 10  Grade 3: 1 | |  |  | 20 (62.50)  Grade 1: n=13  Grade 2: n=6  Grade 3: n=1 | 20 (all at risk) |  | 12 (37.50)  Grade 1: n=10  Grade 2: n=2 | 12 |
| Hypertension in pediatrics |  | Grade 1: 37  Grade 2: 3 | |  |  | 18 (56.25)  Grade 1: n=15  Grade 2: n=3 | 18 (all at risk) |  | 2 (6.25)  Grade 2: n=2 | 2 |

| Hypertriglyceridemia |  | Grade 1: 4  Grade 2: 5  Grade 3: 2 |  |  | 5 (15.63)  Grade 1: n=2  Grade2: n=2  Grade 3: n=1 | 5 (15.63) |  | 3 (9.09)  Grade 1: n=1  Grade 2: n=1  Grade 3: n=1 | 3 |
| --- | --- | --- | --- | --- | --- | --- | --- | --- | --- |
| Left ventricular systolic dysfunction |  | Grade 2: 1 |  |  | 1 (3.13)  Grade 2: n=2 | 1 (3.13)  Grade 2: n=1 |  | 0 |  |
| **CTCAE (449 visits): overall** | | | | **At least once during whole follow-up (n=32)** | | | **Last visit (n=32)** | | |
|  | At risk  n (%) | Outcome  n (%) | At risk **&** outcome  n (%) | At risk  n (%) | Outcome  n (%) | At risk **&** outcome  n (%) | At risk  n (%) | Outcome  n (%) | At risk **&** outcome  n (%) |
| **Endocrine** | 199 (44.32) | 189 (42.09) | 74 | 15 (46.8) | 22 (68.75) | 13 (at risk and outcome) | 14 (43.74) | 17 (53.13) | 7 |
| Abnormal glucose metabolism |  | Grade 3: 2 |  |  | 1 (3.13)  Grade 3: n=1 | 1 (at risk) |  | 0 |  |
| Childhood growth hormone deficiency |  | Grade 1: 3  Grade 2: 12 |  |  | 3 (9.38)  Grade 1: n=1  Grade 2: n=2 | 3 (all at risk) |  | 1 (3.13)  Grade 2: n=1 | 1 |
| Hyperparathyroidism |  | Grade 2: 1 |  |  | 1 (3.13)  Grade 2: n=1 | 0 (pat. with outcome not at risk) |  | 0 |  |
| Hypothyroidism |  | Grade 2: 6 |  |  | 2 (6.26)  Grade 2: n=2 | 2 (all at risk) |  | 1 (3.13)  Grade 2: n=1 | 1 |
| Overweight/Obesity for age ≥ 20 years |  | Grade 2: 33  Grade 3: 9 |  |  | 13 (40.63)  Grade 2: n=9  Grade 3: n=4 | 6 at risk and outcome |  | 11 (34.38)  Grade 2: n=7  Grade 3: n=4 | 4 |
| Overweight/Obesity for age 2 - < 20 years |  | Grade 2: 71  Grade 3: 33 |  |  | 12 (37.50)  Grade 2: n=5  Grade 3: n=7 | 6 at risk and outcome |  | 2 (6.25)  Grade 2: n=1  Grade 3: n=1 | 1 |
| Underweight for age ≥ 20 years |  | Grade 2: 16 |  |  | 4 (12.50)  Grade 2: n=4 | 2 at risk and outcome |  | 1 (3.13)  Grade 2: n=1 | 0 |
| Underweight for age 2 -< 20 years |  | Grade 2: 7 |  |  | 3 (9.38)  Grade 2: n=3 | 2 at risk and outcome |  | 0 |  |
| **CTCAE (449 visits): overall** | | | | **At least once during whole follow-up (n=32)** | | | **Last visit (n=32)** | | |
|  | At risk  n (%) | Outcome  n (%) | At risk **&** outcome  n (%) | At risk  n (%) | Outcome  n (%) | At risk **&** outcome  n (%) | At risk  n (%) | Outcome  n (%) | At risk **&** outcome  n (%) |
| **Gastrointestinal** | 56 (12.47) | 6 (1.34) | 2 | 4 (12.50) | 5 (15.63) | 1 (at risk and outcome) | 4 (12.50) | 0 |  |
| Oesophageal varices |  | Grade 3: 1 |  |  | 1 (3.13)  Grade 3: n=1 | 0 |  | 0 |  |
| Gastritis/duodenitis |  | Grade 2: 4 |  |  | 4 (12.50)  Grade 2: n=4 | 0 |  | 0 |  |
| Pancreatitis |  | Grade 2: 1  Grade 3: 1 |  |  | 1 (3.13)  Grade 3: n=1 | 1 |  | 0 |  |
| **Hepatobiliary** | 449 (100) | 7 (1.56) | 7 (1.56) | 32 (100) | 3 (9.38) | 3 (at risk) | 32 (100) | 0 |  |
| Fibrosis/ Cirrhosis |  | Grade 3: 13  Grade 4: 1 |  |  | 1 (3.13)  Grade 4: n=1 | 1 (at risk) |  | 0 |  |
| Hepatopathy/Alanine and aspartate aminotransferase increased |  | Grade 1: 3 |  |  | 3 (9.38)  Grade 1: n=3 | 3 (at risk) |  | 0 |  |
| Portal hypertension |  | Grade 2: 14 |  |  | 1 (3.13)  Grade 2: n=1 | 1 (at risk) |  | 0 |  |

| **CTCAE (449 visits): overall** | | | | **At least once during whole follow-up (n=32)** | | | **Last visit (n=32)** | | |
| --- | --- | --- | --- | --- | --- | --- | --- | --- | --- |
|  | At risk  n (%) | Outcome  n (%) | At risk AND outcome  n (%) | At risk  n (%) | Outcome  n (%) | At risk AND outcome  n (%) | At risk  n (%) | Outcome  n (%) | At risk **&** outcome  n (%) |
| **Haematologic** | 449(100) | 57 (12.69) | 57 | 32 (100) | 21 (65.63) | 22 | 32 (100) | 4 (12.50) | 4 |
| Anaemia |  | Grade 1: 21  Grade 2: 1 |  |  | 9 (28.13)  Grade 1: n=8  Grade 2: n=1 | 9 (28.13) |  | 2 (6.35)  Grade 1: n=2 | 2 |
| Neutropenia |  | Grade 1: 15  Grade 2: 4  Grade 3: 1 |  |  | 11 (34.38)  Grade 1: n=9  Grade 2: n=1  Grade 3: n=1 | 11 (34.3) |  | 1 (3.13)  Grade 1: n=1 | 1 |
| Polycythaemia |  | Grade 1: 4 |  |  | 3 (9.38)  Grade 1: n=3 | 3 (9.38) |  | 1 (3.13)  Grade 1: n=1 | 1 |
| Thrombocytopenia |  | Grade 1: 7  Grade 2: 5  Grade 3: 11 |  |  | 7 (21.88)  Grade 1: n=6  Grade 3: n=1 | 7 (21.88) |  | 2 (6.25)  Grade 1: n=1  Grade 2: n=1 | 2 |
| **Immunologic** | 37 (8.24) | 2 (0.45) | 3 | 3 (9.38) | 2 (6.25) | 2 (at risk and outcome) | 3 (9.38) | 0 | 0 |
| Graft-versus-host disease |  | Grade 2: 1 |  |  | 1 (3.13)  Grade 2: n=1 | 1 (1.13) |  | 0 |  |
| Immunodeficiency |  | Grade 1: 1 |  |  | 1 (3.13)  Grade 1: n=1 | 0 |  | 0 |  |

| **CTCAE (449 visits): overall** | | | | **At least once during whole follow-up (n=32)** | | | **Last visit (n=33)** | | |
| --- | --- | --- | --- | --- | --- | --- | --- | --- | --- |
|  | At risk  n (%) | Outcome  n (%) | At risk **&** outcome  n (%) | At risk  n (%) | Outcome  n (%) | At risk **&** outcome  n (%) | At risk  n (%) | Outcome  n (%) | At risk **&** outcome  n (%) |
| **Infectious** | 25 (5.79) | 19 (4.23) | 0 | 3 (9.38) | 9 (28.13) | 1 | 1 (3.13) | 0 | 0 |
| Gastrointestinal infect. |  | Grade 2: 2  Grade 3: 1 |  |  | 3 (9.37)  Grade 2: n=2  Grade 3: n=1 | 0 |  |  |  |
| Genitourinary infection |  | Grade 2: 3 |  |  | 3 (9.37)  Grade 2: n=3 | 0 |  |  |  |
| Otitis media, chronic/recurrent |  | Grade 2: 3  Grade 3: 7 |  |  | 2 (6.25)  Grade 2: 1  Grade 3: 1 | 1 |  |  |  |
| Sinusitis, chronic/recurrent |  | Grade 2: 1  Grade 3: 2 |  |  | 1 (3.13)  Grade 3: 1 | 0 |  |  |  |
| **Musculosceletal** | 449 (100) | 109 (23.54) | 109 (24.28) | 32 (100) | 18 (56.25) | 18 (56.25) | 32 (100) | 14 (43.75) | 14 (43.75) |
| Amputation |  | Grade 1: 15  Grade 3: 21 |  |  | 3 (9.38)  Grade 1: n=1  Grade 3: n=2 | 3 (all at risk) |  | 3 (9.38)  Grade 1: n=1  Grade 3: n=2 | 3 |
| Arthralgia |  | Grade 1: 16  Grade 2: 9 |  |  | 5 (15.63)  Grade 1: n=1  Grade 2: n=4 | 5 (all at risk) |  | 2 (6.25)  Grade 1: n=1  Grade 2: n=1 | 2 |
| Bone mineral density deficit in pediatrics |  | Grade 1: n=1  Grade 2: n=1 |  |  | 2 (6.25)  Grade 1: n=1  Grade 2: n=1 | 2 (all at risk) |  | 1 (3.13)  Grade 1: n=1 | 1 |
| Bone mineral density deficit in adults |  | Grade 1: n=13  Grade 2: n=2 |  |  | 10 (31.25)  Grade 1: n=9  Grade 2: n=1 | 10 (all at risk) |  | 7 (21.88)  Grade 1: n=6  Grade 2: n=1 | 7 |
| Dental maldevelopment |  | Grade 1: 15  Grade 2: 1 |  |  | 2 (6.25)  Grade 1: n=1  Grade 2: n=1 | 2 (all at risk) |  | 1 (3.13)  Grade 1: n=1 | 1 |
| Osteonecrosis |  | Grade 1: 6  Grade 2: 9  Grade 3: 16 |  |  | 2 (6.25)  Grade 2: n=1  Grade 3: n=1 | 2 (all at risk) |  | 1 (3.13)  Grade 1: n=1 | 1 |
| Palatal defects, acquired |  | Grade 3: 1 |  |  | 1 (3.13)  Grade 3: n=1 | 1 (all at risk) |  | 0 |  |
| Scoliosis |  | Grade 2: n=8 |  |  | 2 (6.25)  Grade 2: n=2 | 2 (all at risk) |  | 1 (3.13)  Grade 2: n=1 | 1 |
| Temporomandibular joint disorder |  | Grade 2: 12 |  |  | 1 (3.13)  Grade 2: n=1 | 1 (all at risk) |  | 1 (3.13)  Grade 2: n=1 | 1 |
| **CTCAE (449 visits): overall** | | | | **At least once during whole follow-up (n=32)** | | | **Last visit (n=33)** | | |
|  | At risk  n (%) | Outcome  n (%) | At risk **&** outcome  n (%) | At risk  n (%) | Outcome  n (%) | At risk **&** outcome  n (%) | At risk  n (%) | Outcome  n (%) | At risk **&** outcome  n (%) |
| **Neurologic** | 449 (100) | 66 (14.25) | 66 (14.70) | 32 (100) | 9 (28.13) | 9 (27.27) | 32 (100) | 4 (12.12) | 4 |
| Headache (chronic) |  | Grade 1: 22  Grade 2: 14 | 36 |  | 6 (18.75)  Grade 1: n=2  Grade 2: n=4 | 6 (all at risk) |  | 2 (6.25)  Grade 2: n=2 | 2 |
| Paralytic disorders |  | Grade 1: 12 | 12 |  | 1 (3.13)  Grade 1: n=1 | 1 (all at risk) |  | 1 (3.13)  Grade 1: n=1 | 1 |
| Peripheral sensory neuropathy |  | Grade 1: 16 | 16 |  | 2 (6.25)  Grade 1: n=2 | 2 (all at risk) |  | 1 (3.13)  Grade 1: n=1 | 1 |

| **CTCAE (449 visits): overall** | | | | **At least once during whole follow-up (n=32)** | | | **Last visit (n=32)** | | |
| --- | --- | --- | --- | --- | --- | --- | --- | --- | --- |
|  | At risk  n (%) | Outcome  n (%) | At risk **&** outcome  n (%) | At risk  n (%) | Outcome  n (%) | At risk **&** outcome  n (%) | At risk  n (%) | Outcome  n (%) | At risk **&** outcome  n (%) |
| **Ocular, Visual** | 413 (91.98) | 86 (19.15) | 86 (all at risk) | 29 (90.63) | 9 (28.16) | 9 (all at risk) | 30 (93.75) | 7 (30.43) | 7 |
| Cataract |  | Grade 1: 14 |  |  | 2 (6.25)  Grade 1: n=2 | 2 (all at risk) |  | 2 (6.25)  Grade 1: n=2 | 2 |
| Eyelid function disorder |  | Grade 1: 1  Grade 3: 1 |  |  | 1 (3.13)  Grade 3: n=1 | 1 (all at risk) |  | 0 |  |
| Visual acuity, reduced (right exe, OD) |  | Grade 1: 25  Grade 2: 35 |  |  | 6 (18.75)  Grade 1: n=3  Grade 2: n=3 | 6 (all at risk) |  | 5 (15.62)  Grade 1: n=3  Grade 2: n=2 | 5 |
| Visual acuity, reduced (left eye, OS) |  | Grade 1: 25  Grade 2: 35 |  |  | 6 (18.75)  Grade 1: n=3  Grade 2: n=3 | 6 (all at risk) |  | 5 (15.62)  Grade 1: n=3  Grade 2: n=2 | 5 |
| **Pulmonary** | 58 (12.92) | 52 (11.58) | 3 | 4 (12.50) | 5 (15.63) | 1 | 4 (1250) | 5 (15.63) | 0 |
| Asthma |  | Grade 1: 16  Grade 2: 17  Grade 3: 7 |  |  | 3 (9.37)  Grade 1: n=1  Grade 2: n=1  Grade 3: n=1 | 0 |  | 3 (9.37)  Grade 1: n=1  Grade 2: n=2 | 0 |
| Obstructive sleep apnoea |  | Grade 3: 12 |  |  | 1 (3.13)  Grade 13 n=1 | 0 |  | 1 (3.13)  Grade 3: n=1 | 0 |
| Obstructive ventilatory defect (FEV1, FVC) |  | Grade 1: 6  Grade 2: 1 |  |  | 1 (3.13)  Grade 2: n=1 | 0 |  | 0 |  |
| Pulmonary diffusion defect (DLCO) |  | Grade 3: 1 |  |  | 1 (3.13)  Grade 3: n=1 | 1 |  | 0 |  |
| Restrictive ventilatory defect (TLC) |  | Grade 1: 1 |  |  | 1 (3.13)  Grade 1: n=1 | 1 |  | 1 (3.13)  Grade 1: n=1 | 1 |
| **CTCAE (449 visits): overall** | | | | **At least once during whole follow-up (n=32)** | | | **Last visit (n=32)** | | |
|  | At risk  n (%) | Outcome  n (%) | At risk **&** outcome  n (%) | At risk  n (%) | Outcome  n (%) | At risk **&** outcome  n (%) | At risk  n (%) | Outcome  n (%) | At risk **&** outcome  n (%) |
| **Renal, urinary tract** | 463 (100) | 10 (2.16) | 10 (2.16) | 33 (100) | 5 (15.63) | 5 (16.63) | 33 (100) | 3 (9.09) | 3 |
| Chronic haematuria |  | Grade 1: n=5 |  |  | 1 (3.13)  Grade 1: n=1 | 1 |  | 0 |  |
| Chronic kidney disease |  | Grade 1: n=6 |  |  | 4 (12.20)  Grade 1: n=4 | 4 |  | 4 (12.50)  Grade 1: n=4 | 4 |
| Incontinence |  | Grade 1: n=1 |  |  | 1 (3.13)  Grade 1: n=1 | 1 |  | 0 |  |
| **Reproductive, genital** | 463 (100) | 33 (7.35) | 33 (7.35) | 32 (100) | 5 (15.63) | 5 (15.63) | 32 (100) | 4 (12.50) | 4 |
| Dyspareunia |  | Grade 1: n=1 |  |  | 1 (3.13)  Grade 1: n=1 | 1 |  | 1 (3.13)  Grade 1: n=1 | 1 |
| Leydig cell insufficiency |  | Grade 1: n=3  Grade 2: n=14 |  |  | 1 (3.13)  Grade 2: n=1 | 1 |  | 1 (3.13)  Grade 2: n=1 | 1 |
| Primary ovarian failure |  | Present: n=9 |  |  | 1 (3.13) | 1 |  | 1 (3.13) | 1 |
| AMH outside normal range |  | Present: n=2 |  |  | 1 (3.13) | 1 |  | 0 |  |
| Inhibin B outside normal range |  | Present: n=4 |  |  | 1 (3.13) | 1 |  | 1 (3.13) | 1 |

**Supplementary Table 3:** Haematological adverse events less and more than 5 years following completion of treatment

| **Outcome** | **<5 years** (first appearance) | **≥5 years** (first appearance) | **Total** |
| --- | --- | --- | --- |
| **Haematologic** (at risk due to anthracyclines) |  |  | 21 (65.63) |
| Anaemia | Grade 1: n=5  Grade 2: n=1 | Grade 1: n=3 | 9 (28.13)  Grade 1: n=8  Grade 2: n=1 |
| Neutropenia | Grade 1: n=10  Grade 2: n=1 | 0 | 11 (34.38)  Grade 1: n=9  Grade 2: n=1  Grade 3: n=1 |
| Thrombocytopenia | Grade 1: n=5 | Grade 1: n=2 | 7 (21.88)  Grade 1: n=6  Grade 3: n=1 |
